# Equity and gender mainstreaming in public policy: A scoping review protocol

**DOI:** 10.1371/journal.pone.0299124

**Published:** 2024-02-23

**Authors:** Michelle Amri, Jinny Yang, Geneviève Jessiman-Perreault, Amne Haikal, Kathryn Barrett, Jesse B. Bump

**Affiliations:** 1 School of Public Policy, Simon Fraser University, Harbour Center, Vancouver, BC, Canada; 2 Takemi Program in International Health, Harvard T.H. Chan School of Public Health, Harvard University, Boston, MA, United States of America; 3 Dalla Lana School of Public Health, University of Toronto, Toronto, Ontario, Canada; 4 Faculty of Arts, McGill University, Dawson Hall, Montreal, Quebec, Canada; 5 School of Public Policy, University of Calgary, Calgary, AB, Canada; 6 University of Toronto Scarborough Library, Scarborough, ON, Canada; 7 Bergen Centre for Ethics and Priority Setting, University of Bergen, Norway, Bergen, Norway; Johns Hopkins University Bloomberg School of Public Health, UNITED STATES

## Abstract

**Background:**

Despite growing attention paid to health equity and efforts to promote gender mainstreaming—a global strategy to promote gender equality—how policymakers have ‘institutionalized’ this in their work is less clear. Therefore, this planned scoping review seeks to search the peer-reviewed and grey literature to compile evidence on the ways in which policymakers have routinely or systematically considered equity and/or gender in their work.

**Methods:**

A scoping review will be undertaken by drawing on the PRISMA guidelines for Scoping Reviews (PRISMA-ScR). With the expert guidance of a research librarian, Ovid MEDLINE, Ovid EMBASE, PAIS Index, and Scopus databases will be searched, in addition to custom Google searches of government documents. The search will be conducted from 1995 and onwards, as there were no hits prior to this date that included the term “gender mainstream*” in these databases. The inclusion criterion is that: (i) texts must provide information on how equity and/or gender has been considered by government officials in the development of public policy in a routine or systematic manner (e.g., descriptive, empirical); (ii) both texts produced by government or not (e.g., commentary about government action) will be included; (iii) there are no restrictions on study design or article type (i.e., commentaries, reports, and other documents, would all be included); and (iv) texts must be published in English due to resource constraints. However, texts that discuss the work of nongovernmental or intergovernmental organizations will be excluded. Data will be charted by: bibliographic information, including the authors, year, and article title; country the text discussed; and a brief summary on the approach taken.

**Discussion:**

This protocol was developed to improve rigour in the study design and to promote transparency by sharing our methods with the broader research community. This protocol will support a scoping review of the ways in which policymakers have routinely or systematically considered equity and/or gender in their work. We will generate findings to inform government efforts to initiate, sustain, and improve gender and equity mainstreaming approaches in policymaking.

## Introduction

Heightened interest in health equity in recent decades has led to investigations into health equity [1–6] and the uptake of key concepts, including the original Dahlgren-Whitehead rainbow model receiving “worldwide acclaim” [7]. This model, focused on the determinants of health, has been drawn upon in numerous reports focused on health inequalities and has been helpful for policymakers operating outside of the health sector to understand their role in health and health inequities and allowing them to take ownership and responsibility to accordingly act [7]. However, the way policymakers can act in a routine or consistent manner to promote equity in their work is less clear.

Efforts have been undertaken to promote gender “mainstreaming”—a global strategy to promote gender equality [8]—which is in alignment with Sustainable Development Goal 5 on gender equality that seeks to “achieve gender equality and empower all women and girls” [9]. Gender mainstreaming entails considering gender perspectives and aiming for gender equality in all activities, including policy development and decision-making [8]. Gender mainstreaming has been regarded as having “the potential to transform social relations” [10], but at the same time, has been regarded as being “rather hollow when considered within the national setting” [11] and a “mythical beast” [12]. Gender mainstreaming has been noted as being applied across countries in a “technocratic” and “nonsystemic” manner [11]. Thus, the view that gender mainstreaming approaches need to reconsider agency and transformation [12], and more carefully articulate the relationship with societal change [11]. However, there appears to be a gap in knowledge synthesis around how mainstreaming has been carried out.

Therefore, we want to understand precisely how gender mainstreaming, and equity considerations more broadly, have been considered by policymakers globally. As an illustrative example, the Canadian government has adopted Gender-based Analysis Plus, which is an “analytical tool used to support the development of responsive and inclusive policies, programs, and other initiatives” [13]. In doing this analysis, we hope to provide an overview of actions undertaken and related insights that will be helpful for improving public policymaking processes. We seek to search the peer-reviewed and grey literature to compile evidence of approaches to gender mainstreaming and equity considerations more broadly, not focusing in on any specific geography, level of government, or policy domain. Accordingly, we ask the question: “what are the ways in which policymakers have routinely or systematically considered equity and/or gender in their work?” We hope this analysis will also shed light on successes and failures of varying approaches and elucidate if relationships with societal change are explicit. We believe that undertaking this scoping review can result in producing information and guidance for governments to initiate, sustain, and improve such gender and equity mainstreaming approaches in the development of public policy.

## Methods

This scoping review protocol was developed by drawing on the PRISMA-P (Preferred Reporting Items for Systematic review and Meta-Analysis Protocols) [14], PRISMA guidelines for Scoping Reviews (PRISMA-ScR) [15] (as other studies have done [16–20]), and with the expert guidance of a research librarian. The PRISMA/PRISMA-ScR guidelines were followed as they are widely used and can be considered as a best practice tool in evidence syntheses, given that they allow for a largely rigorous and transparent approach to literature searches.

### Eligibility criteria

#### Inclusion criteria

The inclusion criterion is that: (i) texts must provide information on how gender and/or equity has been considered by government officials in the development of public policy in a routine or systematic manner (e.g., descriptive, empirical); (ii) both texts produced by government or not (e.g., commentary about government action) will be included; (iii) there are no restrictions on study design or article type (i.e., commentaries, reports, and other documents, would all be included); and (iv) texts must be published in English due to resource constraints.

#### Exclusion criteria

Texts that solely discuss the work of nongovernmental or intergovernmental organizations will be excluded—please note, this does not apply to nongovernmental texts that observe policy actions within government(s).

### Information sources

**Fig 1:**
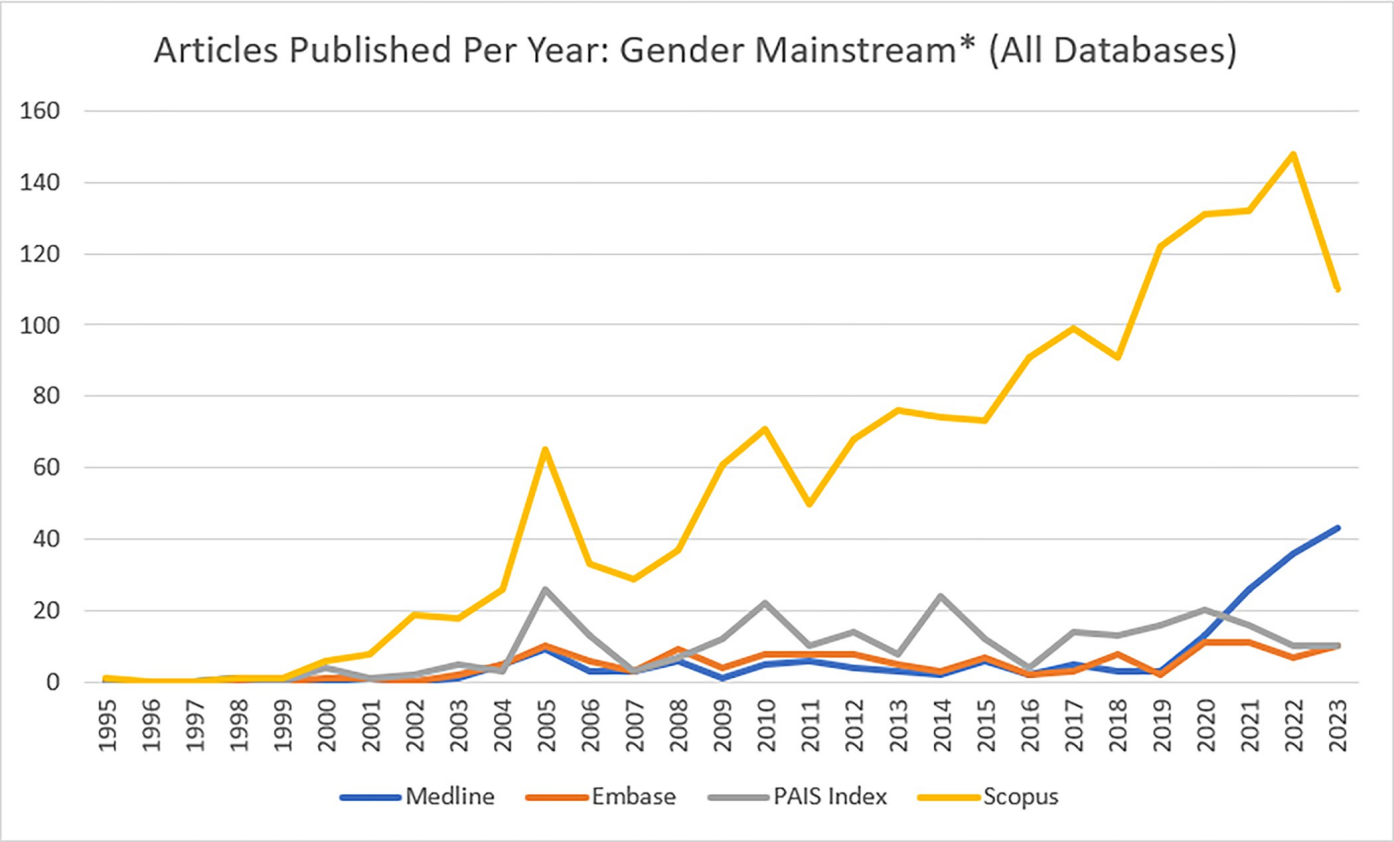
Articles containing the term "gender mainstream*" across four databases.

This study will compile hits from both the peer-reviewed and grey literatures and searches databases from 1995 to the date the search is conducted on. The search will be conducted from 1995 and onwards, as there were no hits prior to this date that included the term “gender mainstream*” in Ovid MEDLINE, Ovid EMBASE, PAIS Index, or Scopus (Fig 1 shows data through the end of 2023, the most recent complete calendar year to maintain comparability).

### Search strategy

Ovid MEDLINE, Ovid EMBASE, PAIS Index, and Scopus databases will be searched to cast a wide net encompassing both biomedical and multidisciplinary databases, complemented with a custom Google search strategy developed by the research librarian to search government documents. The specific rationale for each database and grey literature search, with associated database-specific search strings, are noted in Table 1. In addition to searching for gender mainstreaming, the search strings include gender responsive and gender integrative as terms, given the differing preference for these terms that are synonymous and close in meaning.

**Table 1:** Planned searches, rationale for each database, and associated search strings.

Database	Rationale	Search string or approach
Ovid MEDLINE	Ovid MEDLINE will be searched due to the inclusion of biomedicine and life science journals and serves as a good starting point.	# **Search Statement** 1 Health Equity/ 2 Gender Equity/ 3 ((health adj1 equit$) or (health adj1 inequit$) or (health adj1 equalit$) or (health adj1 inequalit$) or (gender adj1 equit$) or (gender adj1 inequit$) or (gender adj1 equalit$) or (gender adj1 inequalit$)).tw,kf. 4 ((gender adj1 mainstream$) or (gender adj1 integrative) or (gender adj1 responsive) or (equity adj2 mainstream$) or (inequity adj2 mainstream$) or (equality adj2 mainstream$) or (inequality adj2 mainstream$) or (diversity adj2 mainstream$)).tw,kf. 5 1 or 2 or 3 or 4 6 public policy/ or health policy/ 7 Policy Making/ 8 (policy or policies).tw,kf. 9 (policy-mak$ or policy mak$ or policymak$).tw,kf. 10 6 or 7 or 8 or 9 11 5 and 10 12 limit 11 to english language 13 limit 12 to yr = "1995 -Current"
Ovid EMBASE	Ovid EMBASE has a focus on biomedical evidence and has a broad international scope that includes the areas of health policy and public health.	# **Search Statement** 1 health equity/ 2 gender equity/ 3 ((health adj1 equit$) or (health adj1 inequit$) or (health adj1 equalit$) or (health adj1 inequalit$) or (gender adj1 equit$) or (gender adj1 inequit$) or (gender adj1 equalit$) or (gender adj1 inequalit$)).tw,kf. 4 ((gender adj1 mainstream$) or (gender adj1 integrative) or (gender adj1 responsive) or (equity adj2 mainstream$) or (inequity adj2 mainstream$) or (equality adj2 mainstream$) or (inequality adj2 mainstream$) or (diversity adj2 mainstream$)).tw,kf. 5 1 or 2 or 3 or 4 6 public policy/ or health care policy/ 7 (policy or policies).tw,kf. 8 (policy-mak$ or policy mak$ or policymak$).tw,kf. 9 6 or 7 or 8 10 5 and 9 11 limit 10 to english language 12 limit 11 to yr = "1995 -Current"
PAIS Index	The PAIS Index includes international sources and includes a range of public policy materials, including articles, grey literature, research reports, web content, and others.	((((MAINSUBJECT.EXACT("Health") OR MAINSUBJECT.EXACT("Sex")) AND MAINSUBJECT.EXACT("Equity")) OR noft((health NEAR/1 equit*) OR (health NEAR/1 inequit*) OR (health NEAR/1 equalit*) OR (health NEAR/1 inequalit*) OR (gender NEAR/1 equit*) OR (gender NEAR/1 inequit*) OR (gender NEAR/1 equalit*) OR (gender NEAR/1 inequalit*)) OR noft((gender NEAR/1 mainstream) OR (gender NEAR/1 integrative) OR (gender NEAR/1 responsive) OR (equity NEAR/2 mainstream) OR (inequity NEAR/2 mainstream) OR (equality NEAR/2 mainstream) OR (inequality NEAR/2 mainstream) OR (diversity NEAR/2 mainstream))) AND (MAINSUBJECT.EXACT("Public Policy") OR MAINSUBJECT.EXACT("Health Policy") OR MAINSUBJECT.EXACT("Policy Making") OR noft(policy OR policies) OR noft(policy-mak* OR "policy mak*" OR policymak*))) AND pd(19950101–20240112) To apply filter: 1995-01-01 –date searched
Scopus	Scopus will be searched due to its multidisciplinary nature, which includes the fields of medicine and social sciences.	( (TITLE-ABS-KEY ( "health equit*" OR "health inequit*" OR "health equalit*" OR "health inequalit*" OR "gender equit*" OR "gender inequit*" OR "gender equalit*" OR "gender inequalit*" )) OR (TITLE-ABS-KEY ( "gender mainstream*" OR "gender integrative" OR "gender responsive" OR ( equity W/2 mainstream* ) OR ( inequity W/2 mainstream* ) OR ( equality W/2 mainstream* ) OR ( inequality W/2 mainstream* ) OR ( diversity W/2 mainstream* )) ) ) AND ( (TITLE-ABS-KEY ( policy OR policies )) OR (TITLE-ABS-KEY ( policy-mak* OR "policy mak*" OR policymak*) ) ) AND PUBYEAR > 1994 AND PUBYEAR < 2025 AND (LIMIT-TO (LANGUAGE, "English" ))
Government documents	The custom Google search widget can search across specific jurisdictions, which will allow for the inclusion of government reports from around the world.	https://guides.library.utoronto.ca/c.php?g=714472&p=5093475 To use the following custom Google searches: • Canada • United States • Mexico • Africa • Asia and Pacific • Latin America and Caribbean • Europe • Middle East • Distinct searches for “gender mainstream,” “gender mainstreaming,” “gender integrative,” and “gender responsive” will be conducted for each country/region • Will review a minimum of three pages of results (30 items) for all geographies, because there is always at least one relevant title per page for the first three pages of results using this search strategy (with the exception of geographies with fewer results returned) • For each additional page of results, will continue and export all items if there is a minimum of one relevant title on the page

### Study records

#### Data management

Covidence software will be used to compile all texts from the various searches and to eliminate duplicates.

#### Selection process

During the first stage, each text will be screened by reading both the title and abstract for alignment with the inclusion criteria, with each hit reviewed by two reviewers independently. Select authors of the study will serve as reviewers and new reviewers will be added if there are a substantial number of hits to ensure the study remains manageable. Any potential discrepancies will be resolved by a third independent reviewer. Hits screened in through the first stage will be read in-full by two reviewers independently for alignment with the inclusion criteria. For electronic books or other grey literature reports where there are several chapters, potentially relevant chapters will be read in full to determine inclusion. Any potential discrepancies at this stage will be resolved in consultation with a third independent reviewer.

#### Data collection process

After determination of the final hits to be included in the study, data will be extracted from all hits by one researcher. This will be accordingly charted and reviewed by the full authorship team.

#### Data items and charting

The study will chart data from included texts by bibliographic information, including the authors, year, and article title; country the text discussed; type of evidence (e.g., empirical study, government report, commentary); and a brief summary of the approach taken. One reviewer will lead the data charting process and summarizing approaches, which will subsequently be approved by the full authorship team.

#### Outcomes and prioritization

Texts will be coded in NVivo inductively across different types of approaches. These findings will be narratively synthesized to form the results of the study to provide details on each approach. We aspire to also shed light on successes and failures for each approach and assess if these approaches articulate the problematic nature of gender inequity and relationships to systemic societal structures, rather than approach gender mainstreaming and equity considerations in simply a technical manner.

### Limitations

This study is limited by the restriction to English-language results. Excluding other languages may exclude results that would be germane to our study. However, we reasoned that English is by far the dominant language in the professional fields we investigate and therefore our exclusion would have little other no consequence. The exclusion allows us to avoid the biases that would come from interpreting languages not spoken by all members of the research team, as well. The scoping review is also limited by the evidence it assesses. For example, there may be bias in government reports of actions taken. However, we hope the overarching aim of synthesizing evidence will allow for a more fulsome picture of strengths and weaknesses of included studies.

## Conclusion

The development of this protocol serves two key purposes. First, it improves rigour because it sets a priori bounds to maintain a consistent scope of inquiry. It is important to note that setting such rigid bounds early in the study process is designed to lead to limited alterations as the study progresses to reduce cherry-picking of results. However, this can also be restrictive when seeking to report on unpredicted dimensions apparent in retrieved hits. And second, this protocol promotes transparency by publicly documenting our methods. Thus, we hope those interested in this work can benefit from this detailed reporting on our search strategy and approach, along with those designing scoping reviews in adjacent fields.

This protocol will support a scoping review of the ways in which policymakers have routinely or systematically considered equity and/or gender in their work. We will generate findings to inform government efforts to initiate, sustain, and improve gender and equity mainstreaming approaches in policymaking. This is important given persistent gender inequities and the lack of attention to these inequities, notably even in institutions focused on global health, many of which have a central focus in mitigating inequities [21]. And similarly, that the learnings can be applied to multisectoral policy approaches that incorporate or center around health equity, including the Health in All Policies approach and Healthy Cities [22]. This is of particular importance in light of COVID-19 potentially affording an opportunity to refocus on health equity [23] and drawing attention to the need to focus on equity outcomes, instead of prioritizing fiscal considerations [24].

## Supporting information

S1 Checklist Preferred Reporting Items for Systematic reviews and Meta-Analyses extension for Scoping Reviews (PRISMA-ScR) checklist. (DOCX)

## Abbreviations

PRISMA-P: Preferred Reporting Items for Systematic review and Meta-Analysis Protocols
PRISMA-ScR: PRISMA guidelines for Scoping Reviews
